# Evaluation of the Potency of the Anti-Idiotypic Antibody Ab2/3H6 Mimicking gp41 as an HIV-1 Vaccine in a Rabbit Prime/Boost Study

**DOI:** 10.1371/journal.pone.0039063

**Published:** 2012-06-15

**Authors:** Alexander Mader, Renate Kunert

**Affiliations:** Department of Biotechnology, VIBT - BOKU – University of Natural Resources and Life Sciences (Vienna), Vienna, Austria; Tulane University, United States of America

## Abstract

The HIV-1 envelope protein harbors several conserved epitopes that are recognized by broadly neutralizing antibodies. One of these neutralizing sites, the MPER region of gp41, is targeted by one of the most potent and broadly neutralizing monoclonal antibody, 2F5. Different vaccination strategies and a lot of efforts have been undertaken to induce MPER neutralizing antibodies but little success has been achieved so far. We tried to consider the alternative anti-idiotypic vaccination approach for induction of 2F5-like antibodies. The previously developed and characterized anti-idiotypic antibody Ab2/3H6 was expressed as antibody fragment fusion protein with C-terminally attached immune-modulators and used for immunization of rabbits to induce antibodies specific for HIV-1. Only those rabbits immunized with immunogens fused with the immune-modulators developed HIV-1 specific antibodies. Anti-anti-idiotypic antibodies were affinity purified using a two-step affinity purification protocol which revealed that only little amount of the total rabbit IgG fraction contained HIV-1 specific antibodies. The characterization of the induced anti-anti-idiotypic antibodies showed specificity for the linear epitope of 2F5 GGGELDKWASL and the HIV-1 envelope protein gp140. Despite specificity for the linear epitope and the truncated HIV-1 envelope protein these antibodies were not able to exhibit virus neutralization activities. These results suggest that Ab2/3H6 alone might not be suitable as a vaccine.

## Introduction

Currently 33 million people are living with human immunodeficiency virus type 1 (HIV-1) worldwide. In 2009 2.6 million people became newly infected and 1.8 million people died in the course of AIDS [1]. During the last decades several efforts to induce HIV-1 defending neutralizing antibodies (Abs) have failed [2]–[4] but also promising results were reported [5], [6]. One of the most potent neutralizing HIV-1 Abs isolated so far is the monoclonal Ab (mAb) 2F5 [7]–[11] which binds to the membrane proximal external region (MPER) of the virus envelope glycoprotein gp41 [12], [13]. The potency of such neutralizing Abs alone and in combination was demonstrated by passive immunization and viral challenge in non-human primate models [14]–[17]. Therefore the specific induction of likewise broadly neutralizing Abs against the MPER, 2F5-like Abs, is a major goal for Ab-based HIV-1 vaccine strategies. Despite a strong humoral response to gp41 during the course of HIV-1 infection is evident [18], approaches to elicit cross-clade neutralizing Abs against the MPER region were difficult to achieve [19]–[21].

An alternative method to induce neutralizing Abs is the anti-idiotypic (Id) approach. This approach is based on the idiotype network theory postulated by Jerne about the Ab (Ab1) - anti-Id Ab (Ab2) – anti-anti-Id Ab (Ab3) cascade stimulation, whereby specific anti-Id Abs can serve as an "internal image" of the target antigen and can be used to induce Ab3s that can bind to the cognate antigen [22]. Anti-Id Abs have been proposed as vaccines for cancer immunotherapy and significant success has been achieved using anti-Id vaccines mimicking tumor-associated antigens in animal studies [23]–[26] as well as in clinical trails [27]. The anti-Id Ab Ab2/3H6 was developed at the Department of Biotechnology [28] and is directed against mAb 2F5. The chimeric as well as humanized version of Ab2/3H6 significantly inhibits the binding of mAb 2F5 to its synthetic epitope ELDKWA in an equimolar ratio and also decreases the *in vitro* neutralization potency of mAb 2F5 in a dose-related manner [29]–[31]. Ab2/3H6 is therefore estimated to mimic the epitope of mAb 2F5 and would be of great therapeutic interest as an anti-Id HIV-1 vaccine. To improve the potency of the anti-Id Ab we designed fusion proteins consisting of Ab2/3H6 Ab fragments (Ab2/3H6^Fab^) and C-terminally attached polypeptides to induce T-cell responses against the virus.

One molecule with a wide range of biological activities is the immune stimulatory cytokine interleukin 15 (IL15). It is involved in the activation and proliferation of CD8+ T-cells and natural killer T-cells, the maintenance of CD8+ memory cells, and the differentiation and maturation of B cells [32], [33]. Previous studies have shown that the incorporation of IL15 into vaccinia-based smallpox vaccine [34] or tuberculosis vaccine [35] induces high avidity, long lived antigen specific memory T-cells as well as persistent antigen specific Ab responses.

Other interesting immunostimulatory peptides are the so-called “promiscuous” T-cell epitopes from tetanus toxin (TT), measles virus, or E6 transforming protein [36], [37]. It has been proposed that T-cells provide “help” to B cells under genetic control which can be provided by incorporation into an effective vaccine. Previous studies showed that co-immunization of the consensus cavealin-1 binding domain peptide with the T-cell epitope from TT increased the production of HIV-1 neutralizing Abs in a macaque prime/boost study [38].

Therefore we recombinantly expressed fusion proteins of Ab2/3H6^Fabs^ with the IL15 and alternatively an epitope of TT, respectively. In this study we immunized rabbits with the different Ab2/3H6^Fabs^ and evaluated the humoral immune response as well as the neutralization potency of the obtained Ab3s to those generated with Ab2/3H6^Fab^ only.

## Materials and Methods

### Ethics Statement

Serological tests of rabbits were performed at the Institute of Small Farm Animals, Animal Production Research Center, Nitra (Slovakia) in accordance with Slovak and EU regulations concerning animal experiments (Slovak national law 115/1995). Study approval was issued based on the study protocol by ethics committee of the Institute of Small Farm Animals, Animal Production Research Centre Nitra under the approval ID SK P 28004.

### Expression Vectors

The Ab2/3H6^Fab^ expression vectors containing the murine variable regions of Ab2/3H6 (ABP04229, ABP04230) and the human constant CH1 and Ckappa regions were described previously [29]. The coding region of the heavy chain (HC) genes of Ab2/3H6^Fab^-IL15 and Ab2/3H6^Fab^ –TT were cloned into pcDNA3 (Invitrogen) containing the CMV promoter and the neomycin phospho-transferase gene (neo). pIRESdhfr [39] was used as backbone for the coding region of the Ab2/3H6 light chain (LC). The genes of Ab2/3H6^Fab^-IL15 HC and Ab2/3H6 LC were synthesized by Geneart (Regensburg, Germany) including the *Gaussia* luciferase signal peptide [40] for both, the HC and LC. The sequence of the human IL15 (P40933) gene was obtained from the UniProtKB/Swiss-Prot database. The TT epitope (YSYFPSV) [41] was introduced by PCR amplification of Ab2/3H6^Fab^ HC sequence with the sense primer 5′- accctggtgaccgtgtcc-3′ and antisense primer 5′- aggagcggccgcctatacagatggaaaatatgaataggcagatcctcctccgcc-3′ containing a *NotI* restriction site for exchange of the IL15 tag.

### Expression of Ab2/3H6^Fab^ Variants in CHO Cells

Stable cell lines were generated by co-transfection of corresponding HC and LC plasmids into CHO dhfr negative cells (ATCC CRL-9096; [42]). Clones were selected with Geneticin G418 (Fisher Scientific) and methotrexate (MTX) (Sigma) in combination with limiting dilution subcloning. Collected supernatants were concentrated by the Stirred cell 8200 with UF Discs Ultracel RC 10 kD (Millipore) before purification.

### Purification of Ab2/3H6^Fab^ Variants

Purification of all Ab2/3H6^Fab^ variants was performed on a BioLogic Duo Flow chromatography system (Biorad). Concentrated cell culture supernatant was diluted 1∶2 in buffer A (20 mM Na_2_HPO_4_, pH 7.2) and filtered through a 0.22 µm syringe filter (Millipore). The UNO Q 1.3 ml anion exchange (AIX) column (Biorad) was equilibrated with 5 column volumes (cv) buffer A. Five millilitre of the concentrated sample were loaded onto the column using direct injection via the DuoFlow F40 pump at a flow rate of 1 ml/min corresponding to a linear velocity of 155.9 cm/h. The column was washed with buffer A until the optical density returned to base line. Ab2/3H6^Fab^s were eluted from the AIX column using a 3 step gradient of 20, 40 and 100% of buffer B (20 mM Na_2_HPO_4_, 1 M NaCl pH 7.2) in buffer A in 5 cv at a flow rate of 1.0 ml/min (155.9 cm/h). Ten microlitres samples from the eluted AIX chromatography fractions were analysed by SDS-PAGE and NN-silver stain. Quantification of Ab2/3H6^Fab^ was done by double sandwich enzyme-linked immunosorbent assay (ELISA). Ab2/3H6^Fab^ containing fractions were further purified on a Superdex 75 prep grade Tricorn 10/300 column (GE) with PBS as mobile phase at a flow rate of 0.5 ml/min, corresponding to a linear velocity of 38.2 cm/h.

### Animals and Immunization

Ab2/3H6^Fab^, -IL15, and -TT preparations were used for the immunisation of New Zealand white rabbits. Six rabbits (groups of two rabbits per preparation) were immunised subcutaneously and intramuscularly with 0.1 mg of purified Ab2/3H6^Fab^ proteins emulsified in complete Freund’s adjuvant and boosted two times with the same Fab preparations in incomplete Freund’s adjuvant at three-week intervals.

### Purification of Antibody Fractions from Crude Sera

#### Purification of rabbit IgG

Purification of rabbit IgG from crude sera was performed on an Äkta Purifier chromatography system (GE) with the UNOsphere SUPrA Mini cartridge column (Biorad). IgGs were eluted from the Protein A column using a step gradient of 80% of buffer B (100 mM Glycin pH 3.5) in buffer A (100 mM Glycin, 100 mM NaCl, pH 7.0). Quantification of rabbit IgG was done by double sandwich ELISA.

#### Immunoaffinity purification of gp140/ELDKWA specific Ab3s

HIV-1 specific Ab3s were purified from rabbit IgG fractions using an affinity column coupled with recombinant gp140 (HIV-1 cladeA, 92/UG/037), named UG37 [43] and the synthetic mAb 2F5 epitope (GGGELDKWASL). The affinity matrix was prepared with the AminoLink Plus Immobilization Kit (Thermo Scientific) following the manufacturer’s instructions. Briefly, a mixture of 1.5 mg UG37 combined with 0.5 mg GGGELDKWASL (both Polymun Scientific, Inc) was coupled to the beads using the pH 10 coupling procedure. Pooled rabbit IgG fractions were diluted 1∶2 with binding buffer and 2 ml batches were incubated with the conjugated beads for 1h at RT. The eluted fractions were pooled, concentrated using Amicon Ultra-15 3k columns (Millipore) and named Ab3 pool.

### Evaluation of Humoral Immune Response

#### ELISA

Rabbit IgG concentration and specificity for Ab2/3H6^Fab^ and HIV-envelope were analysed by ELISA. The 96-well microtiter plates (Nunc) were coated over night at 4°C with 1 µg/mL of anti-rabbit IgG (Sigma), Ab2/3H6^Fab^, UG37 or GGGELDKWASL peptide, respectively. Plates were blocked with PBS containing 3% BSA and 0.1% Tween 20 in PBS (RT, 1 h) and incubated with serial twofold dilutions of heat inactivated serum samples or purified rabbit IgG (RT, 1 h). Afterwards plates were incubated with peroxidase-conjugated anti-rabbit IgG antibody (Sigma) and reactions were visualised with o-phenylenediamine and H_2_O_2_ (Merck). Cut-off values were estimated from ELISA using the two-fold OD value of the negative control.

#### Affinity analysis

Bio-Layer Interferometry (BLI) was used for determination of binding affinity of Ab3s and 2F5 single chain/fragment crystallisable fusion protein (2F5scFc) at a starting concentration of 400 nM. Kinetic measurements were performed with the Octet QK (fortéBio). Streptavidin (SA) biosensor tips were loaded with 13 µg/mL of biotinylated UG37 or biotinylated GGGELDKWASL peptide according to the manufacturer’s instructions. The assay was performed at 30°C in PBS buffer with 1000 rpm agitation. Association and dissociation curves (5 min each) were recorded for the individual samples and data were processed and analyzed using the Octet data analysis software 6.4 (fortéBio).

#### Competition assay

BLI was used for competition experiments as describes elsewhere [44]. Biotinylated GGGELDKWASL peptide or biotinylated UG37 was coupled onto SA biosensor tips. Antigen coated tips were dipped into wells of 2F5scFc or the Ab3 pool (each at 400 nM, 5 min) until saturation, and then moved into wells containing the “competing” Ab (Ab3s or 2F5scFc; 400 nM, 5 min).

#### Neutralization assay

Pseudotyped virions were generated in HEK293T (ATCC: CRL-11268) cells by co-transfection with HIV-1 Env plasmid SF162 (NIHARRRP; contributed by L. Stamatatos and C. Cheng-Mayer) and the HIV-1 Env-deleted backbone plasmid pSG3ΔEnv (NIHARRRP; contributed by J. Kappes and X. Wu), as previously described [45]. Virions were added to the same volume of serially diluted mAbs (starting at 25 µg/mL) and incubated for 1 h at 37°C. Then, freshly trypsinized TZM-bl reporter cells (NIHARRRP; contributed by J. Kappes and X.Wu) (10,000 cells in 100 µL growth medium supplemented with DEAE-dextran at a final concentration of 10 µg/mL) were added and the plates were incubated at 37°C. After 48 h incubation, the medium was removed and 50 µL lysis buffer (25 mM glycylglycin, 15 mM MgSO4, 4 mM EDTA, 1% Triton X-100 in H2O, pH 7.8) was added. Finally, 50 µL Bright-Glo reagent (Promega) was added and luminescence was measured using a luminometer (Tecan). All experiments were performed at least in duplicate. The IC50 was calculated with GraphPad Prism 5.04 (GraphPad Software, Inc.) using a four parameter fit.

## Results

### Expression and Purification of Ab2/3H6^Fab^ Fusion Proteins

**Figure 1:**
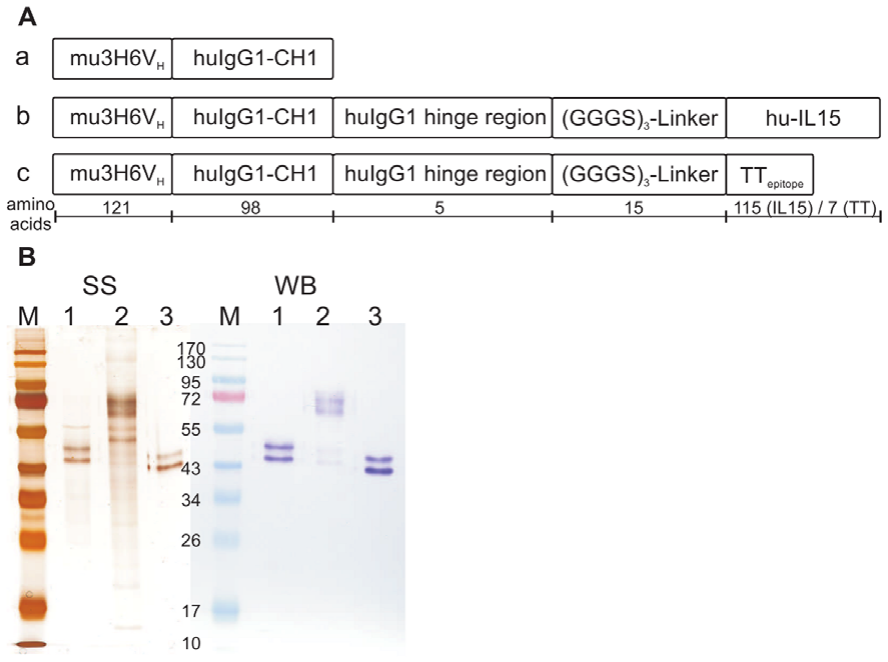
Design and purification of Ab2/3H6^Fab^ variants. Three different Ab2/3H6^Fab^ preparations were developed for the immunization study. Panel **A.** displays (**a**) the Fab-fragment HC of Ab2/3H6 named Ab2/3H6^Fab^. (**b**) human IL15 fused to Ab2/3H6^Fab^ named Ab2/3H6^Fab^-IL15 and (**c**) a tetanus toxin epitope fused to Ab2/3H6^Fab^ named Ab2/3H6^Fab^-TT. The chimeric mouse 3H6vL/hukappa LC (214 aa) is not shown. Panel **B.** shows the non-reduced SDS-gel of purified Fabs; silver stain (SS) on the left side and the Western Blot (WB) on the right side developed using an anti-human Fab specific antibody and visualized with NBT/BCIP. The lanes 1 represents Ab2/3H6^Fab^-TT (49 kD); lane 2: Ab2/3H6^Fab^-IL15 (61 kD); lane 3: Ab2/3H6^Fab^ (47 kD) and M represents the marker. The double band is significant for glycosylated Ab2/3H6 [61].

Ab2/3H6^Fab^ fusion proteins (Figure 1A) were expressed in CHO cells by cotransfecting pAb2/3H6^Fab^-IL15 or pAb2/3H6^Fab^-TT in combination with pAb2/3H6LC. The previously developed recombinant cell line expressing Ab2/3H6^Fab^ [29] was used for purification of Ab2/3H6^Fab^. Culture supernatants containing approximately two mg of each of the recombinant Ab2/3H6^Fab^ variants were purified on a Q-Sepharose column followed by a size-exclusion step. Purified proteins were quantified by ELISA, and analyzed by SDS-PAGE and Western Blot (Figure 1B).

### Humoral Immune Response in Rabbits

New Zealand white rabbits were immunized with three different Ab2/3H6^Fab^ variants in duplicates in a prime/boost regime. Ten days after the final boost animals were bled to death and serum was collected. Afterwards IgG was purified from rabbit sera to avoid nonspecific binding of accompanied serum proteins. Pre-immune sera and immune sera of all immunized animals as well as sera of the two control rabbits were screened for total rabbit IgG content ranging from 9 to 22 mg/ml per animal. Immunization with the IL15 or TT fusion protein did not increase total IgG levels significantly compared to immunization with Ab2/3H6^Fab^ (data not shown). Samples from individual rabbits were coded accordingly: Fab-1 and Fab-2 for Ab2/3H6^Fab^ immunized animals; IL15-1 and IL15-2 for Ab2/3H6^Fab^-IL15 immunized animals, TT-1 and TT-2 for Ab2/3H6^Fab^-TT immunized animals and N-1 and N-2 for control rabbits.

#### Specificity against Ab2/3H6^Fab^ and HIV-1 epitopes

**Figure 2:**
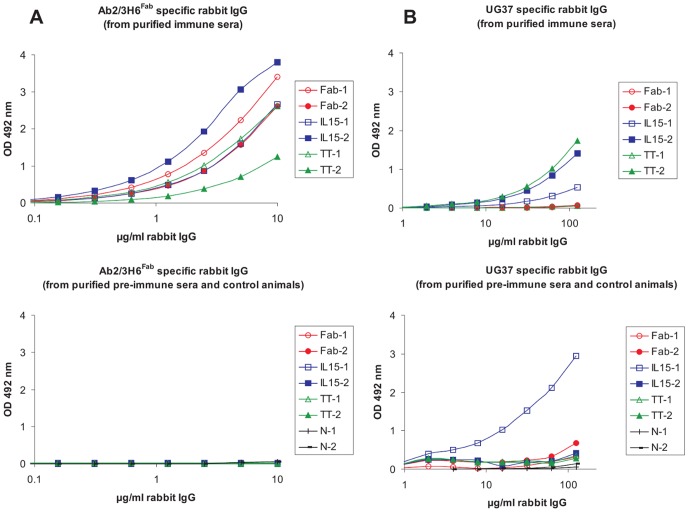
Binding of different immunized rabbit IgG fractions to Ab2/3H6^Fab^ and UG37. Purified rabbit IgG fractions immunized with different Fab preparations, the pre-immune IgG fractions and the IgG fractions of the control animals were tested in a binding ELISA. Diluted IgG fractions from individually immunized rabbits binding to Ab2/3H6^Fab^ (**A**; upper panel) and associated pre-immune/control fractions (**A**; lower panel) as well as diluted IgG fractions from individually immunized rabbits binding to UG37 (**B**; upper panel) and associated pre-immune/control fractions (**B**; lower panel) are blotted against the OD-value. (Samples codes: Fab for Ab2/3H6^Fab^, IL15 for Ab2/3H6^Fab^-IL15 and TT for Ab2/3H6^Fab^-TT immunized rabbits).

The specificity of IgG fractions to the Ab2/3H6^Fab^ antigen was estimated in an ELISA starting with a concentration of 10 µg/ml rabbit IgG. Fab-1, Fab-2, IL15-1, IL15-2 and TT-1 samples show a similar binding curve resulting in cut-off concentrations between 0.2 µg/ml and 0.5 µg/ml whereas the TT-2 sample (cut-off 1.4 µg/ml) shows weaker binding (Figure 2A; upper panel). Rabbit IgG of purified pre-immune sera as well as control animals showed no binding towards Ab2/3H6^Fab^ (Figure 2A; lower panel).

Additionally, the IgG fractions were tested on HIV-1 envelope protein UG37 coated ELISA plates with a starting concentration of 125 µg/ml rabbit IgG. The rabbit IgG samples IL15-1, IL15-2 and TT-2 showed significant specificity to UG37 as shown in Figure 2B (upper panel) with cut-off concentrations of 21, 7 and 6 µg/ml. Fab-1, Fab-2 and TT-1 did not bind to UG37 despite the immunized rabbits developed antibodies with high specificity for the immunogen Ab2/3H6^Fab^. One of the pre-immune samples (from animal IL15-1) bound to UG37 (Figure 2B; lower panel). This pre-immune fraction exhibits a 5-fold stronger binding to UG37 than the corresponding immune-fraction. The reason for this unexpected result is not clear.

The statistical significance between immunized and non-immunized rabbits or rabbits immunized with the immune-modulator fusion proteins and Ab2/3H6^Fab^ was analysed using the Fisher’s Exact test (p<0.05).

The calculated p-value for the induction of anti-Ab2/3H6^Fab^ Abs is 0.0003 (Table 1) indicating a strong significant correlation between immunized and non-immunized animals. The p-value for the induction of anti-UG37 Abs is 0.4000 (Table 1) indicating no statistical significance using Ab2/3H6^Fab^ Abs with or without immune-modulators.

**Table 1:** Fisher’s Exact test.

Ab2/3H6^Fab^ binding	immunized rabbits	pre-immune sera and control animals	Total
**YES**	6	0	6
**NO**	0	8	8
**Total**	6	8	14

UG37 binding	rabbits immunized with immune-modulators	rabbits immunized without immune-modulators	Total
**YES**	3	1	4
**NO**	0	2	2
**Total**	3	3	6

Fisher’s Exact test two-tailed.

p = 0.0003.

p>0.05 (highly statistically significant).

#### Characterization of the immunoaffinity purified Ab3 pool

**Figure 3:**
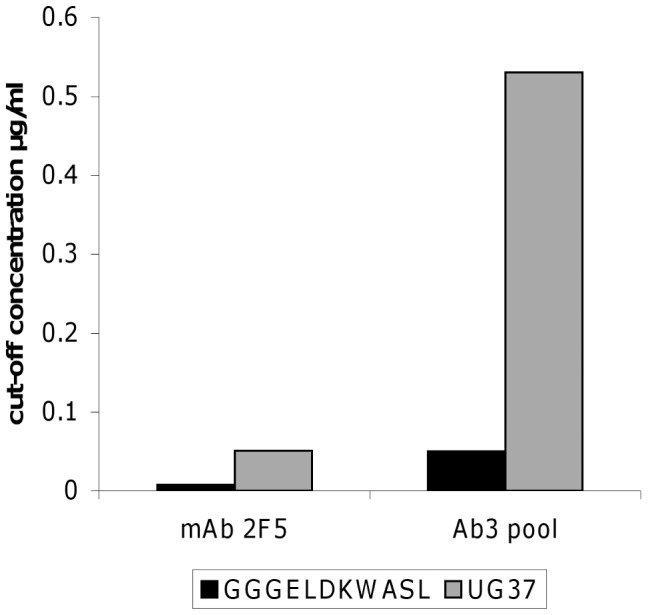
Cut-off values of the Ab3 pool binding to GGGELDKWASL and UG37. Affinity purified rabbit Ab3 pool was tested in a binding ELISA for GGGELDKWASL and UG37 specificity. Cut-off values were calculated for 2-fold the OD-value of the non immunized control animal sera.

Pooled rabbit IgG samples IL15-2 and TT-2 were purified with a second affinity purification step, using a UG37/GGGELDKWASL coupled column. The results of the affinity purification step are summarized in Table 2 and show that only 1.5% of the rabbit IgG fraction contained Ab3s. The collected Ab3 pool was tested for specificity to UG37 and the linear epitope GGGELDKWASL starting with 1 µg/ml rabbit IgG in ELISA. UG37 and GGGELDKWASL specificity was estimated by cut-off values (Figure 3) indicating approximately 10-fold stronger binding of the Ab3 fraction to the linear epitope GGGELDKWASL than to UG37. In comparison mAb 2F5 exhibits a seven-fold weaker binding to UG37 than to GGGELDKWASL (Figure 3).

**Table 2:** Mass balance of Ab3 affinity purification.

	total IgG [mg]	Yield [% of rabbit IgG]
**rabbit IgG**	8.00	–
**Flow through**	5.54	69.2
**Wash**	2.29	28.7
**Ab3 pool**	0.12	1.5

The total rabbit IgG recovery after each run was approximately 99%.

**Figure 4:**
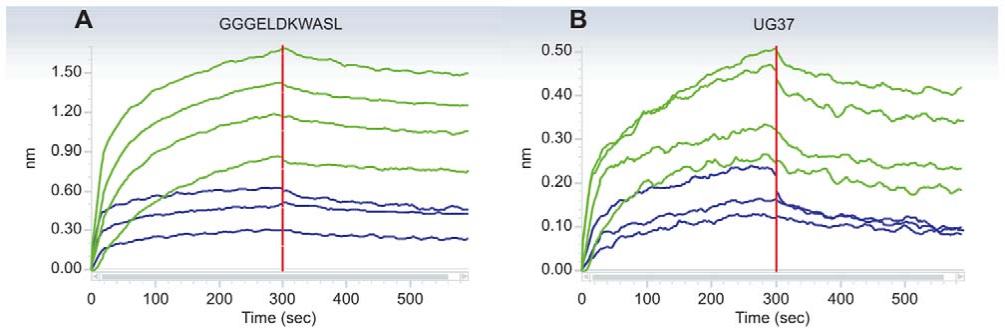
Affinity measurement of Ab3 pool to GGGELDKWASL and UG37. Affinity purified Ab3 pool and 2F5scFc were tested in a BLI assay for binding affinity to GGGELDKWASL and UG37. Binding curves of mAb 2F5scFc (green) and Ab3 pool (blue) are displayed. Each antibody was applied with a starting concentration of 400 nM and was further two-fold diluted in a dilution row. The data was fitted using the 1∶1 model (curve-fit not shown).

We confirmed the ELISA binding studies with affinity analysis using BLI. GGGELDKWASL or UG37 coated sensor tips were used for affinity measurements of the specific Ab3 pool. Sample and 2F5scFc as positive control were measured in decreasing concentrations (400, 200, 100 and 50 nM). Figure 4A (GGGELDKWASL biosensor tips) and Figure 4B (UG37 biosensor tips) display the association and dissociation curves, indicating specific binding and dissociation of the induced Ab3s to both GGGELDKWASL and UG37. The binding properties shown by the k_on_, k_off_ and K_D_ values of 2F5scFc and the Ab3 pool to GGGELDKWASL are comparable meaning similar affinity of 2F5scFc and Ab3 pool to the synthetic epitope while the affinity of the Ab3 pool to UG37 is five-fold lower compared to 2F5scFc (Table 3).

**Table 3:** Summary of k-values for binding to HIV-1 epitopes.

	mAb 2F5scFc		Ab3 fraction	
	UG37	GGGELDKWASL	UG37	GGGELDKWASL
**kon [1/Ms]**	1.9×10^05^ (1.3)	1.2×10^05^ (0.4)	8.4×10^04^ (3.4)	1.9×10^05^ (0.5)
**koff [1/s]**	9.5×10^−04^ (3.2)	3.2×10^−04^ (1.1)	2.3×10^−03^ (1.3)	6.6×10^−04^ (3.5)
**KD [nM]**	7.1 (4.3)	2.7 (0.3)	33.3 (28.3)	3.6 (2.4)

standard deviation in brackets.

#### Competition assay

**Figure 5:**
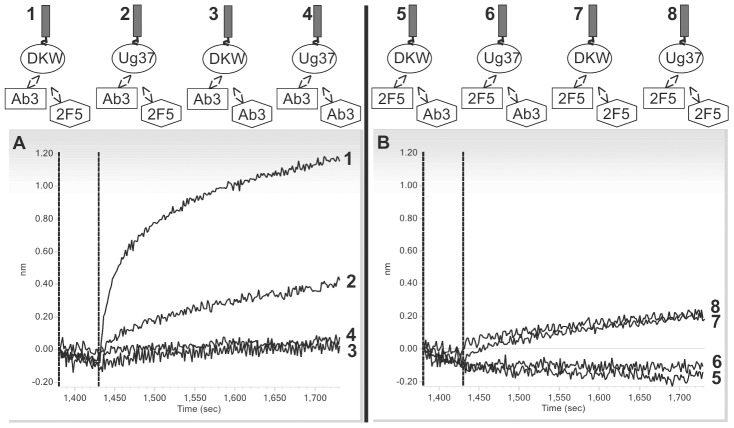
Competition curves of Ab3 pool and mAb 2F5scFc to the epitopes GGGELDKWASL and UG37. A (upper panel) shows the assay setup of 2F5scFc competing with the Ab3 pool for the epitopes of GGGELDKWASL and UG37. In the lower panel the competition curves of mAb 2F5scFc to the Ab3 pool are displayed. **B** (upper panel) shows the assay setup of the Ab3 pool competing with 2F5scFc for the epitopes of GGGELDKWASL and UG37. In the lower panel the competition curves of the Ab3 pool to mAb 2F5scFc are displayed.

Competition tests were generated using the BLI method to verify the specificity of the induced Ab3s to GGGELDKWASL and UG37. The assay setup is shown in Figure 5 (upper panel). GGGELDKWASL or UG37 saturated tips were incubated with the Ab3 pool. For competition the sensor tips were transferred to a solution of 2F5scFc or the Ab3s for self-competition as control. The obtained competition curves indicate that 2F5scFc is able to displace the Ab3 pool from both epitopes (GGGELDKWASL and UG37) (Figure 5A). In a second assay 2F5scFc was allowed to bind to GGGELDKWASL or UG37 tips until saturation. Afterwards the saturated sensor tips were transferred to a solution of the Ab3 pool for competition or 2F5scFc for self-competition as control. The recorded competition curves showed that the Ab3 fraction was not able to compete with 2F5scFc (Figure 5B).

#### Neutralization assay

To investigate the neutralization potency of the purified gp140 specific Ab3 pool a TZM-bl Env-pseudotyped virus assay against the HIV-1 SF162 Env clone was performed. In this assay mAb 2F5 inhibits entry of the pseudotyped virus with an IC_50_ of 130 ng/ml. The purified Ab3s induced by immunization of rabbits with Ab2/3H6 shows no neutralization activity (data not shown).

## Discussion

The global HIV pandemic is still expanding and thus the development of a preventive vaccine is of high priority. One approach is to elicit broadly neutralizing Abs against the MPER region that resembles similar potency as the mAb 2F5. In previous studies MPER-containing proteins [46], [47] or MPER-containing peptides [48]–[50] failed to elicit broadly neutralizing Abs, presumably due to the poor immunogenicity of the MPER [21]. It was also suggested that the native gp41 exodomain is structurally more complex than represented by the linear epitope [51], [52] and thus incorrect conformation of MPER-based peptide immunogens result in suboptimal presentation of neutralizing epitopes [53], [54]. Additionally, a so far unidentified part of a second epitope has been proposed [55] to interact with the long CDR-H3 loop of mAb 2F5. This could be an alpha-helix C-terminal of the core epitope DKW [56] or membrane compounds from the infected cells [57]–[59]. Such a rather complex neutralizing epitope calls for an alternative vaccine approach and we decided to continue with anti-Id antibodies. In previous studies Ab2/3H6 showed promising results mimicking the gp41 epitope of mAb 2F5. Gach et al. demonstrated that Ab2/3H6 is able to inhibit the binding of mAb 2F5 to its synthetic epitope ELDKWA and to induce Ab3s (2F5-like Abs) in a mouse immunization study [29], [30]. But due to the small amount of mouse sera obtained more extensive characterizations could not be performed. Co-crystallization of Ab2/3H6 in complex with mAb 2F5 and comparison with epitope/mAb 2F5 co-crystals revealed that Ab2/3H6 only partly overlaps with the HIV-1 epitope on mAb 2F5 but does not center on the core epitope binding site of mAb 2F5. Thus Ab2/3H6 was classified as a gamma-class anti-Id Ab [60]. However, since mAb 2F5 crystals were generated with a small peptide of the MPER only, the exact binding mechanism of mAb 2F5 to HIV remains elusive and the most critical paratope responsible for the neutralization activity of mAb 2F5 might not be identified yet.

In this study we aimed to induce 2F5-like Abs using an anti-Id network approach, instead of using MPER epitopes to elicit neutralizing Abs. We constructed Fab fusion proteins of Ab2/3H6 containing the molecular adjuvant IL15 or a “promiscuous” T-cell epitope of TT and administered them in a rabbit prime/boost regime. Total rabbit IgG levels, Ab2/3H6^Fab^ and specificity for HIV-1 epitopes were measured in an ELISA showing that the use of IL15 and TT as immune stimulators do not significantly influence total rabbit IgG levels (data not shown). However, after protein A purification we were able to detect UG37 binding Ab3s (Figure 2B) in three out of six animals. Interestingly these three animals were immunized using fusion proteins of Ab2/3H6^Fab^ with immune-modulators.

This observation could not be approved statistically (Table 1) due to the small test group used and the fact that one animal immunized with immune-modulator fusion protein did not show binding to UG37. We strongly believe that by increasing the number of animals tested the specificity and the advantage of immune-modulators for induction of Ab3s might also be confirmed statistically. In the next step we immunoaffinity purified selected rabbit IgG samples to enrich the induced Ab3s using a UG37/GGGELDKWASL coupled column with a rather low recovery of 1.5% of the rabbit IgG fraction (Table 2). This immunoaffinity enriched Ab3 pool shows significant binding to GGGELDKWASL and also considerable interaction with UG37 (Figure 3 and 4). But affinities were lower compared to the 2F5scFc; approximately five-times lower for UG37 and slightly lower for GGGELDKWASL (Table 3). Competition data revealed that the purified Ab3 pool is not able to displace 2F5scFc for its epitopes but on the other hand 2F5scFc replaces the Ab3 pool from its binding to GGGELDKWASL and UG37 (Figure 5A and 5B). The lack of competition ability of the Ab3 pool is probably based on the higher affinity of 2F5scFc for its epitopes. Since the neutralization assays failed to indicate that neutralizing Abs were generated in rabbits, we hypothesize a second epitope that is responsible for the neutralization potency of mAb 2F5. Additionally, the long H3 loop of mAb 2F5 indicates intensive somatic mutagenesis and it is questionable if a seven week prime/boost schedule in rabbits can reflect the maturation of the human immune system.

To conclude on this study, Ab2/3H6 in combination with immune-modulators like IL15 or TT was capable to induce anti-anti-idiotypic Abs against the synthetic epitope of mAb 2F5 as well as the HIV-1 envelope protein UG37. Despite the induced Ab3s were not able to inhibit infection of TZM-bl cells in an Env-pseudotyped neutralization assay Ab2/3H6 at least mimics part of the epitope. Affinity and competition data proved their specificity but weaker binding of the induced Ab3s compared to mAb 2F5 is evident.

After decades of intense research regarding the induction of 2F5-like Abs a potent immunogen that is able to induce broadly neutralizing 2F5-like Abs is still missing. Deciphering the complete and correct mechanism on how mAb 2F5 prevents gp41 to fuse with the host cell membrane is the major task in developing HIV-1 vaccines able to induce broadly neutralizing anti-MPER Abs. Unfortunately, the anti-Id approach using Ab2/3H6 does not lead to the desired outcome.

## References

[1] HIV/AIDS UJPo (2010). Global Report: UNAIDS Report on the Global AIDS Epidemic.

[2] Plotkin S (2008). Vaccines: correlates of vaccine-induced immunity.. Clin Infect Dis.

[3] Amanna IJ, Slifka MK (2009). Wanted, dead or alive: new viral vaccines.. Antiviral Res.

[4] Zinkernagel RM (2003). On natural and artificial vaccinations.. Annu Rev Immunol.

[5] Nishiyama Y, Planque S, Mitsuda Y, Nitti G, Taguchi H (2009). Toward effective HIV vaccination: induction of binary epitope reactive antibodies with broad HIV neutralizing activity.. J Biol Chem.

[6] Ye L, Wen Z, Dong K, Wang X, Bu Z (2011). Induction of HIV Neutralizing Antibodies against the MPER of the HIV Envelope Protein by HA/gp41 Chimeric Protein-Based DNA and VLP Vaccines.. PLoS One.

[7] Buchacher A, Predl R, Strutzenberger K, Steinfellner W, Trkola A (1994). Generation of human monoclonal antibodies against HIV-1 proteins; electrofusion and Epstein-Barr virus transformation for peripheral blood lymphocyte immortalization.. AIDS Res Hum Retroviruses.

[8] Purtscher M, Trkola A, Gruber G, Buchacher A, Predl R (1994). A broadly neutralizing human monoclonal antibody against gp41 of human immunodeficiency virus type 1.. AIDS Res Hum Retroviruses.

[9] Mehandru S, Wrin T, Galovich J, Stiegler G, Vcelar B (2004). Neutralization profiles of newly transmitted human immunodeficiency virus type 1 by monoclonal antibodies 2G12, 2F5, and 4E10.. J Virol.

[10] Kunert R, Steinfellner W, Purtscher M, Assadian A, Katinger H (2000). Stable recombinant expression of the anti HIV-1 monoclonal antibody 2F5 after IgG3/IgG1 subclass switch in CHO cells.. Biotechnol Bioeng.

[11] Kunert R, Rüker F, Katinger H (1998). Molecular characterization of five neutralizing anti-HIV type 1 antibodies: identification of nonconventional D segments in the human monoclonal antibodies 2G12 and 2F5.. AIDS Res Hum Retroviruses.

[12] Stoiber H, Frank I, Spruth M, Schwendinger M, Mullauer B (1997). Inhibition of HIV-1 infection in vitro by monoclonal antibodies to the complement receptor type 3 (CR3): an accessory role for CR3 during virus entry?. Mol Immunol.

[13] Stiegler G, Kunert R, Purtscher M, Wolbank S, Voglauer R (2001). A potent cross-clade neutralizing human monoclonal antibody against a novel epitope on gp41 of human immunodeficiency virus type 1.. AIDS Res Hum Retroviruses.

[14] Mascola JR, Stiegler G, VanCott TC, Katinger H, Carpenter CB (2000). Protection of macaques against vaginal transmission of a pathogenic HIV-1/SIV chimeric virus by passive infusion of neutralizing antibodies.. Nat Med.

[15] Veazey RS, Shattock RJ, Pope M, Kirijan JC, Jones J (2003). Prevention of virus transmission to macaque monkeys by a vaginally applied monoclonal antibody to HIV-1 gp120.. Nat Med.

[16] Hessell AJ, Rakasz EG, Poignard P, Hangartner L, Landucci G (2009). Broadly neutralizing human anti-HIV antibody 2G12 is effective in protection against mucosal SHIV challenge even at low serum neutralizing titers.. PLoS Pathog.

[17] Hessell AJ, Rakasz EG, Tehrani DM, Huber M, Weisgrau KL (2010). Broadly neutralizing monoclonal antibodies 2F5 and 4E10 directed against the human immunodeficiency virus type 1 gp41 membrane-proximal external region protect against mucosal challenge by simian-human immunodeficiency virus SHIVBa-L.. J Virol.

[18] Opalka D, Pessi A, Bianchi E, Ciliberto G, Schleif W (2004). Analysis of the HIV-1 gp41 specific immune response using a multiplexed antibody detection assay.. J Immunol Methods.

[19] Hu SL, Stamatatos L (2007). Prospects of HIV Env modification as an approach to HIV vaccine design.. Curr HIV Res.

[20] Karlsson Hedestam GB, Fouchier RA, Phogat S, Burton DR, Sodroski J (2008). The challenges of eliciting neutralizing antibodies to HIV-1 and to influenza virus.. Nat Rev Microbiol.

[21] Montero M, van Houten NE, Wang X, Scott JK (2008). The membrane-proximal external region of the human immunodeficiency virus type 1 envelope: dominant site of antibody neutralization and target for vaccine design.. Microbiol Mol Biol Rev 72: 54–84, table of contents.

[22] Jerne N (1974). Towards a network theory of the immune system.. Ann Immunol (Paris).

[23] Ladjemi MZ, Chardes T, Corgnac S, Garambois V, Morisseau S (2011). Vaccination with human anti-trastuzumab anti-idiotype scFv reverses HER2 immunological tolerance and induces tumor immunity in MMTV.f.huHER2(Fo5) mice.. Breast Cancer Res.

[24] Ramos AS, Parise CB, Travassos LR, Han SW, de Campos-Lima PO (2011). The idiotype (Id) cascade in mice elicited the production of anti-R24 Id and anti-anti-Id monoclonal antibodies with antitumor and protective activity against human melanoma.. Cancer Sci.

[25] Lee G, Ge B (2010). Inhibition of in vitro tumor cell growth by RP215 monoclonal antibody and antibodies raised against its anti-idiotype antibodies.. Cancer Immunol Immunother.

[26] Wang JJ, Li YH, Liu YH, Song J, Guo FJ (2010). The ability of human bispecific anti-idiotype antibody to elicit humoral and cellular immune responses in mice.. Int Immunopharmacol.

[27] de Cerio AL, Zabalegui N, Rodríguez-Calvillo M, Inogés S, Bendandi M (2007). Anti-idiotype antibodies in cancer treatment.. Oncogene.

[28] Kunert R, Weik R, Ferko B, Stiegler G, Katinger H (2002). Anti-idiotypic antibody Ab2/3H6 mimics the epitope of the neutralizing anti-HIV-1 monoclonal antibody 2F5.. AIDS.

[29] Gach J, Quendler H, Weik R, Katinger H, Kunert R (2007). Partial humanization and characterization of an anti-idiotypic antibody against monoclonal antibody 2F5, a potential HIV vaccine?. AIDS Res Hum Retroviruses.

[30] Gach J, Quendler H, Strobach S, Katinger H, Kunert R (2008). Structural analysis and in vivo administration of an anti-idiotypic antibody against mAb 2F5.. Mol Immunol.

[31] Mader A, Kunert R (2010). Humanization strategies for an anti-idiotypic antibody mimicking HIV-1 gp41.. Protein Eng Des Sel.

[32] Waldmann TA (2006). The biology of interleukin-2 and interleukin-15: implications for cancer therapy and vaccine design.. Nat Rev Immunol.

[33] Rodrigues L, Bonorino C (2009). Role of IL-15 and IL-21 in viral immunity: applications for vaccines and therapies.. Expert Rev Vaccines.

[34] Perera LP, Waldmann TA, Mosca JD, Baldwin N, Berzofsky JA (2007). Development of smallpox vaccine candidates with integrated interleukin-15 that demonstrate superior immunogenicity, efficacy, and safety in mice.. J Virol.

[35] Kolibab K, Yang A, Derrick SC, Waldmann TA, Perera LP (2010). Highly persistent and effective prime/boost regimens against tuberculosis that use a multivalent modified vaccine virus Ankara-based tuberculosis vaccine with interleukin-15 as a molecular adjuvant.. Clin Vaccine Immunol.

[36] Panina-Bordignon P, Tan A, Termijtelen A, Demotz S, Corradin G (1989). Universally immunogenic T cell epitopes: promiscuous binding to human MHC class II and promiscuous recognition by T cells.. Eur J Immunol.

[37] Kaumaya PT, Kobs-Conrad S, Seo YH, Lee H, VanBuskirk AM (1993). Peptide vaccines incorporating a ‘promiscuous’ T-cell epitope bypass certain haplotype restricted immune responses and provide broad spectrum immunogenicity.. J Mol Recognit.

[38] Benferhat R, Martinon F, Krust B, Le Grand R, Hovanessian AG (2009). The CBD1 peptide corresponding to the caveolin-1 binding domain of HIV-1 glycoprotein gp41 elicits neutralizing antibodies in cynomolgus macaques when administered with the tetanus T helper epitope.. Mol Immunol.

[39] Kunert R, Wolbank S, Stiegler G, Weik R, Katinger H (2004). Characterization of molecular features, antigen-binding, and in vitro properties of IgG and IgM variants of 4E10, an anti-HIV type 1 neutralizing monoclonal antibody.. AIDS Res Hum Retroviruses.

[40] Knappskog S, Ravneberg H, Gjerdrum C, Trösse C, Stern B (2007). The level of synthesis and secretion of Gaussia princeps luciferase in transfected CHO cells is heavily dependent on the choice of signal peptide.. J Biotechnol.

[41] Ho PC, Mutch DA, Winkel KD, Saul AJ, Jones GL (1990). Identification of two promiscuous T cell epitopes from tetanus toxin.. Eur J Immunol.

[42] Urlaub G, Chasin L (1980). Isolation of Chinese hamster cell mutants deficient in dihydrofolate reductase activity.. Proc Natl Acad Sci U S A.

[43] Jeffs SA, Goriup S, Kebble B, Crane D, Bolgiano B (2004). Expression and characterisation of recombinant oligomeric envelope glycoproteins derived from primary isolates of HIV-1.. Vaccine.

[44] Abdiche YN, Malashock DS, Pinkerton A, Pons J (2009). Exploring blocking assays using Octet, ProteOn, and Biacore biosensors.. Anal Biochem.

[45] Li M, Gao F, Mascola JR, Stamatatos L, Polonis VR (2005). Human immunodeficiency virus type 1 env clones from acute and early subtype B infections for standardized assessments of vaccine-elicited neutralizing antibodies.. J Virol.

[46] Zhang MY, Wang Y, Mankowski MK, Ptak RG, Dimitrov DS (2009). Cross-reactive HIV-1-neutralizing activity of serum IgG from a rabbit immunized with gp41 fused to IgG1 Fc: possible role of the prolonged half-life of the immunogen.. Vaccine.

[47] Kim M, Qiao Z, Yu J, Montefiori D, Reinherz EL (2007). Immunogenicity of recombinant human immunodeficiency virus type 1-like particles expressing gp41 derivatives in a pre-fusion state.. Vaccine.

[48] Wang Z, Liu Z, Cheng X, Chen YH (2005). The recombinant immunogen with high-density epitopes of ELDKWA and ELDEWA induced antibodies recognizing both epitopes on HIV-1 gp41.. Microbiol Immunol.

[49] Joyce JG, Hurni WM, Bogusky MJ, Garsky VM, Liang X (2002). Enhancement of alpha -helicity in the HIV-1 inhibitory peptide DP178 leads to an increased affinity for human monoclonal antibody 2F5 but does not elicit neutralizing responses in vitro. Implications for vaccine design.. J Biol Chem.

[50] Ho J, MacDonald KS, Barber BH (2002). Construction of recombinant targeting immunogens incorporating an HIV-1 neutralizing epitope into sites of differing conformational constraint.. Vaccine.

[51] Lorizate M, Cruz A, Huarte N, Kunert R, Pérez-Gil J (2006). Recognition and blocking of HIV-1 gp41 pre-transmembrane sequence by monoclonal 4E10 antibody in a Raft-like membrane environment.. J Biol Chem.

[52] Menendez A, Chow KC, Pan OC, Scott JK (2004). Human immunodeficiency virus type 1-neutralizing monoclonal antibody 2F5 is multispecific for sequences flanking the DKW core epitope.. J Mol Biol.

[53] Ofek G, Tang M, Sambor A, Katinger H, Mascola J (2004). Structure and mechanistic analysis of the anti-human immunodeficiency virus type 1 antibody 2F5 in complex with its gp41 epitope.. J Virol.

[54] Cardoso R, Zwick M, Stanfield R, Kunert R, Binley J (2005). Broadly neutralizing anti-HIV antibody 4E10 recognizes a helical conformation of a highly conserved fusion-associated motif in gp41.. Immunity.

[55] Julien JP, Bryson S, Nieva JL, Pai EF (2008). Structural details of HIV-1 recognition by the broadly neutralizing monoclonal antibody 2F5: epitope conformation, antigen-recognition loop mobility, and anion-binding site.. J Mol Biol.

[56] Bryson S, Julien JP, Hynes RC, Pai EF (2009). Crystallographic definition of the epitope promiscuity of the broadly neutralizing anti-human immunodeficiency virus type 1 antibody 2F5: vaccine design implications.. J Virol.

[57] Alam SM, Morelli M, Dennison SM, Liao HX, Zhang R (2009). Role of HIV membrane in neutralization by two broadly neutralizing antibodies.. Proc Natl Acad Sci U S A.

[58] Scherer EM, Leaman DP, Zwick MB, McMichael AJ, Burton DR (2010). Aromatic residues at the edge of the antibody combining site facilitate viral glycoprotein recognition through membrane interactions.. Proc Natl Acad Sci U S A.

[59] Ofek G, McKee K, Yang Y, Yang ZY, Skinner J (2010). Relationship between antibody 2F5 neutralization of HIV-1 and hydrophobicity of its heavy chain third complementarity-determining region.. J Virol.

[60] Bryson S, Julien J, Isenman D, Kunert R, Katinger H (2008). Crystal structure of the complex between the F(ab)’ fragment of the cross-neutralizing anti-HIV-1 antibody 2F5 and the F(ab) fragment of its anti-idiotypic antibody 3H6.. J Mol Biol.

[61] Gach JS, Maurer M, Hahn R, Gasser B, Mattanovich D (2007). High level expression of a promising anti-idiotypic antibody fragment vaccine against HIV-1 in Pichia pastoris.. J Biotechnol.

